# Autoimmunity in Rheumatic Diseases Is Induced by Microbial Infections via Crossreactivity or Molecular Mimicry

**DOI:** 10.1155/2012/539282

**Published:** 2012-02-20

**Authors:** Taha Rashid, Alan Ebringer

**Affiliations:** Analytical Sciences Group, Kings College London, 150 Stamford Street, London SE1 9NN, UK

## Abstract

A general consensus supports fundamental roles for both genetic and environmental, mainly microbial, factors in the development of autoimmune diseases. One form of autoimmune rheumatic diseases is confined to a group of nonpyogenic conditions which are usually preceded by or associated with either explicit or occult infections. A previous history of clinical pharyngitis, gastroenteritis/urethritis, or tick-borne skin manifestation can be obtained from patients with rheumatic fever, reactive arthritis, or Lyme disease, respectively, whilst, other rheumatic diseases like rheumatoid arthritis (RA), ankylosing spondylitis (AS), and Crohn's disease (CD) are usually lacking such an association with a noticeable microbial infection. A great amount of data supports the notion that RA is most likely caused by *Proteus* asymptomatic urinary tract infections, whilst AS and CD are caused by subclinical bowel infections with *Klebsiella* microbes. Molecular mimicry is the main pathogenetic mechanism that can explain these forms of microbe-disease associations, where the causative microbes can initiate the disease with consequent productions of antibacterial and crossreactive autoantibodies which have a great impact in the propagation and the development of these diseases.

## 1. Introduction

The exact triggering factor in most autoimmune diseases is unknown, yet an infectious cause has long been suggested to have an important role in the development of autoimmunity. Many epidemiologic and clinical reports show a prompt increase in the incidence of several immune-mediated disorders, such as rheumatoid arthritis (RA), inflammatory bowel disease (IBD), and primary biliary cirrhosis in the Western populations throughout the world. This rapid rise in the number of autoimmune diseases cannot be explained solely on the basis of genetic association, but also through the involvement of exogenous (environmental) factors predominantly in the form of microbial infections [1]. In this review, we are discussing the role of microbes in some immune-mediated rheumatologic disorders, such as rheumatic fever, Lyme disease, reactive arthritis (ReA), RA, ankylosing spondylitis (AS), and Crohn's disease (CD).

## 2. Interplay of Genetic and Environmental Factors in the Development of RA, AS, and CD

It is generally agreed that genetics form the main components of the aetiologic factors in the development of autoimmune diseases. For example, more than 95% of patients with AS possess HLA-B27, a class I major histocompatibility complex (MHC) gene, whilst its frequency among the general population is less than 10% [2, 3]. So far, this relationship is considered as the most powerful genetic-disease association holding true for many different populations [4]. Meanwhile, the frequency of HLA-B27 allelotypes in CD patients without associated arthritis is usually the same as in the normal population, but it is increased to up to 60% in those with involvement of the spinal joints [5].

In RA, however, class II MHC gene, HLA-DR4, is the most strongly linked genetic marker to this disease. The frequency of this allelotype has been found to be around 70% in RA patients but it is detected in less than 30% of the general population [6]. A homologous molecular structure, consisting of a glutamic acid, glutamine, arginine, arginine, alanine, and alanine “EQRRAA” amino acid sequence (present in some subtypes of HLA-DR*β*1 molecules, such as HLA-DR*β*1*0401 and HLA-DR*β*1*0404, but not in others such as HLA-DR*β*1*0402) has been described in patients with RA and was given the name of “shared epitope” [7]. The frequency of the “shared epitope” was found to be increased to more than 90% in patients with RA [8].

The high associations between genetic haplotypes and these diseases cannot explain the considerably low incidence of these conditions among the relatives and even twins of patients with these diseases. For example, the concordance rate, the chance of the second twin of developing the disease, in monozygotic twins was found to be 40% in AS [9], 15% in RA [10], and less than 14% in CD patients [11], which indicates that other nongenetic environmental, probably microbial, factors are also involved in the aetiopathogenesis of these conditions.

## 3. Evidence for Associated or Preceding Bacterial Infections in Some Rheumatic Diseases

Extensive evidence supports the role for microbial infections in the development of various rheumatic diseases. The infection whether being associated with or preceding these conditions usually takes either an overt or occult form. Furthermore, these rheumatic disorders are usually associated with certain elements of autoimmune features in the form of elevated levels of autoantibodies to systematically distributed or organ-specific tissues. Certain examples of these microbe-triggered immune-mediated rheumatic disorders are discussed below.

### 3.1. Rheumatic Diseases Following Overt Bacterial Infections

#### 3.1.1. Rheumatic Fever and Streptococcal Infections

Rheumatic fever is the prototype of postinfectious rheumatologic conditions following upper respiratory tract infections by group A beta hemolytic streptococcus pyogenes. This disease is considerably commoner in developing countries and its incidence may reach up to 50 per 100,000 [12]. Apart from involving the joints with a classical migratory polyarthritis, this condition is also characterized by other nonmusculoskeletal features which are induced by pathological lesions involving the heart (rheumatic carditis), and brain (Sydenham's chorea) [13]. Patients with rheumatic fever showing any of these clinical presentations are usually investigated for the evidence of previous infections by this microbe.

Apart from increased levels of anti-streptolysin O titres, serological analysis in these patients will also show elevated levels of auto-antibodies against the M protein and carbohydrate antigens which are crossreactive with the streptococcal antigens and expressed on the tissues of joints [14], heart [15], and basal ganglia in brain [16].

In a recent study, it has been found that passive immunization with anti-streptococcal exotoxin B monoclonal antibodies which also bind cardiac endothelial cells have caused IgG deposition, complement activation, and apoptotic cell death in the experimental mouse heart valve [17]. In the same study, it was shown that the binding ability of these monoclonal antibodies to the endothelial cells was blocked significantly by pretreatment with crossreactive amino acid peptide sequences taken from N-Acetyl-*β*-D-glucosamine, when conjugated with bovine serum albumin (BSA), but not with BSA alone.

#### 3.1.2. Lyme Disease and Spirochetal Infections

Lyme disease or borreliosis, the most common vector-borne illness in the United States, is a multisystemic disorder caused by infections with spirochete microbes, *Borrelia burgdorferi*, and transmitted via Ixodes (deer) tick bites [18]. In this disease, different clinical stages with their probable explanations have been recognized [19]. Patients with Lyme disease are usually presented with or give a past history of localized skin manifestations in the form of well-characterized cutaneous rash and itching described as “erythema migrans” with or without a flu-like illness resulting from the tick bite. After a considerable period of time, patients with this illness show disseminated or wide-spread features due to the involvement of the cardiac, nervous, and musculo-skeletal systems as the result of infections by the causative microbes and/or their associated antimicrobial immune responses. The later phases of this illness, referred to as chronic Lyme disease and post-Lyme disease syndrome, are characterized by persistent arthritic and neurological features occurring as the result of the tissue damages induced by the effects of the cross-reacting antibodies to spirochetal and self-antigens [20, 21].

#### 3.1.3. Reactive Arthritis and Its Association with Enteropathic and Uropathogenic Bacterial Infections

Reactive arthritis (ReA) is included as one component of a group of several inter-related but phenotypically different disorders that are collectively named as “spondylarthritis” (SpA) which comprises AS, psoriatic arthritis, undifferentiated SpA, and IBD consisting of two subsets; CD and ulcerative colitis [22].

It was in the early twentieth century when a link between infection and the occurrence of a triad of urethritis, conjunctivitis and arthritis (Reiter's syndrome) was discovered [23]. Reiter's syndrome was later recognized as a form of ReA which has commonly been associated with explicit preceding infections by enteropathic microbial agents including *Campylobacter*, *Salmonella*, *Shigella* and *Yersinia,* as well as the uropathogenic, *Chlamydia* bacteria [24].

In a most recent study from Finland, ReA was identified in 21 out of 45 referred patients suspected of having the disease after an extensive sewage contamination of the water supply system in the town of Nokia. Enteropathic microbial agents, including *Campylobacter*, *Yersinia,* and *Salmonella,* were isolated in 33% of these patients. These findings indicate that mere exposures to infections are not enough and the interplay of genetic and other susceptibility factors play a role in the disease pathogenesis [25].

The pathogenetic mechanism in this disease can be explained on the basis that secretory antibodies against these microbes which are produced in the gut are transferred into the joint spaces where they bind tissues expressing crossreactive self-antigens such as HLA-B27 molecules [26, 27].

### 3.2. Rheumatic Diseases Associated with Occult or Hidden Bacterial Infections

#### 3.2.1. Evidence of Immunological, Molecular, and Microbiological Link between *Proteus* and RA

Since the mid 1980s, extensive efforts including many studies have emphasized a role of *Proteus mirabilis* microbes in the aetiopathogenesis of RA. Briefly, evidence for the role of *Proteus* in the initiation and development of RA can be summarized as follows.

**Figure 1:**
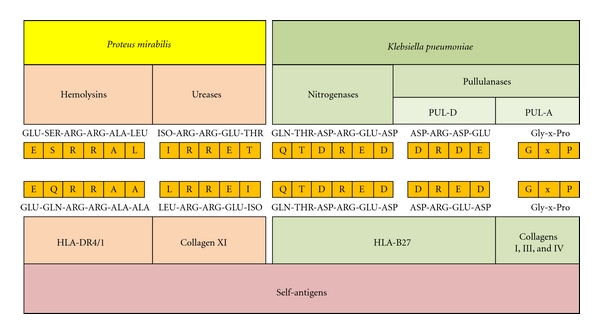
Schematic representation of the molecular similarities between bacterial, *Klebsiella* or *Proteus, *and self-antigens.

- Rabbits injected with HLA-DR4-positive lymphocytes were found to produce antibodies which will only bind to *P. mirabilis* but not 18 other microbes [28].
- Tissue-typing sera from pregnant women having anti-HLA-DR4 specificity were found to bind more significantly to *P. mirabilis* than to *E. coli* [29].
- Molecular similarities were found between “ESRRAL” amino acid motifs present in the hemolysins enzyme products of *Proteus* microbes and “EQRRAA” molecular sequences present in HLA-DR4/1 haplotypes [30]. Furthermore, “IRRET” amino acid motifs expressed on surface antigens of *Proteus* urease enzymes were found to be homologous to “LRREI” molecular sequences present in type XI collagen fibres (Figure 1) [31].
- Significant reciprocal bindings were detected between “EQRRAA” synthetic peptides and ESRRAL anti-sera raised in rabbits, and also between “ESRRAL” peptides with anti-EQRRAA antibodies from immunized rabbits. Furthermore, anti-ESRRAL peptide antibodies were found to bind preferably to mouse fibroblast transfectant cell line expressing HLA-DR*β*1*0401, containing EQRRAA sequence, but not to HLA-DR*β*0402, lacking EQRRAA sequence [32]. These results clearly indicate that antibodies to the “shared epitope” have tissue binding activity.
- IgG antibodies from patients with RA were found to have cytotoxic activities against HLA-DR4-peptide-bearing cells as shown by increased haemolysis for the sheep red blood cells coated with HLA-DR*β*1*0404 peptides when compared to sera from AS and healthy control subjects [27].
- Several independent groups have found that antibodies to *P. mirabilis* microbes were significantly elevated in patients with RA compared to those with other diseases or corresponding healthy subjects recruited from 15 different countries throughout the world (Table 1) [33, 34].
- Evidence for the microbiological link between RA and *Proteus* microorganisms are mainly based on the findings of a group from Scotland, where the isolation rates of *P. mirabilis *bacteria from urine samples of patients with RA were found to be twice as high as that of *E. coli* [35]. A similar result was previously reported by our group from England, where *Proteus* microbes were isolated more significantly in female (63%) and male (50%) patients with RA than healthy women (32%) and men (11%) control subjects [36]. Moreover, urine samples from patients with RA were also shown to contain elevated levels of antibodies to *P. mirabilis* [35], and a positive correlation was found between the levels of these antibodies in sera and urine samples of RA patients [37]. Further evidence has come from results of previous studies, where patients with RA had increased incidence of urinary tract infections [38, 39].

These immunological, molecular, and microbiological findings support the notions that there is a crucial role for *Proteus* microorganisms in the initiation and perpetuation of RA. Furthermore, evidence exists which indicate that in RA *Proteus* infections usually occur in subclinical or asymptomatic forms [40].

#### 3.2.2. Evidence of Immunological, Molecular, and Microbiological Link between *Klebsiella* and AS

The roles of *Klebsiella pneumonia* pathogens in the aetiopathogenesis of AS are mainly based on results of many studies which have been carried by several independent groups. These results can be summarized as follows.

- Sera from rabbits immunized with lymphocytes expressing HLA-B27 haplotypes were binding significantly to antigenic extracts of *Klebsiella* but not to those of other microbes [43]. Anti-HLA-B27 allogeneic human tissue typing sera were found to bind more preferably to *Klebsiella* microbes in comparison to other HLA-specific antisera [44].
- HLA-B27 monoclonal antibodies were found to bind *Klebsiella*, *Shigella,* and *Yersinia* enterobacteria indicating the existence of some crossreactive antigens in these microbes [45]. Other anti-HLA-B27 monoclonal antibodies, however, were found to bind *Klebsiella* more preferably than *Shigella* and *Yersinia* microbial antigens [46].
- Molecular similarities, comprising a hexameric amino acid sequence; glutamine, threonine, aspartic acid, arginine, glutamic acid, and aspartic acid, “QTDRED,” have been found between *Klebsiella* nitrogenase reductase enzymes and HLA-B27 self-antigen molecules [47]. A quadrimeric homologous structure was also found to exist in both *Klebsiella* pullulanase pul-D secretion proteins, comprising; aspartic acid, arginine, aspartic acid, and glutamic acid, “DRDE” molecules and HLA-B27 haplotype, comprising aspartic acid, arginine, glutamic acid, and aspartic acid, “DRED” molecules, as well as a similarity which involves repeated molecular motifs, consisting of glycine-x-proline (Gly-x-Pro) amino acid sequences present in both *Klebsiella* pullulanase Pul-A secretion proteins and collagen type I, III, and IV fibres (Figure 1) [48].
- Antisera from immunized rabbits with *Klebsiella* were found to bind equally to B27-positive lymphocytes whether obtained from AS patients or healthy controls but not to lymphocytes taken from HLA-B27-negative individuals [49]. This indicates that there is no immunological discrepancy between surface antigens of both diseased and normal HLA-B27 molecules when treated with anti-*Klebsiella* antibodies.
- *Klebsiella* antibodies were significantly elevated in the serum compared to the synovial fluid of AS patients [50], indicating that these antibodies are produced in extra-articular regions such as the enteric mucosal lymphatic system before gaining entry into the joints.
- Extensive immunological studies have been carried out during the last three decades by different independent groups throughout the world. The results of these studies indicate that antibodies against *K. pneumoniae* and/or crossreactive self-antigens but not against other microbial agents are significantly elevated among patients with AS when compared to patients with other diseases or to healthy individuals (Table 1) [41, 42].
- IgG antibodies from AS patients were found to possess significant *in vitro* cytotoxic activities to HLA-B27 peptide-bearing cells when compared to RA patients or healthy subjects, when they showed increased percentage lysis of the sheep red blood cells coated with HLA-B27 peptides containing the crossreactive antigens [27].
- Antibodies to *Klebsiella* nitrogenase “QTDRED-” containing peptides were found to bind the synovial tissues of AS patients more significantly when compared to those from patients with other rheumatic diseases [51].
- Microbiological evidence for a link between *Klebsiella* microorganisms and AS are mainly based on the results of various studies, where increased isolation rates of *Klebsiella* from the bowel of AS patients have been reported to correlate with disease activity status [52–55]. Other groups, however, could not find such an association [56, 57]. These discrepancies in the results could be explained by the differences in the method of collection and culture of the faecal specimens and the disease activity status. Furthermore, in patients with AS elevated levels of IgA *Klebsiella* antibodies were found to be associated with a higher degree of gut inflammation [58] and the source of these bacterial antibodies were shown to be the jejunal region of the gut [59].
- HLA-B27 transgenic rats raised in a germ-free environment do not develop many features of SpAs, particularly the gut and arthritic lesions, which may indicate that the commensal gut flora plays an important role in the pathogenesis of B27-associated arthropathies [60].

The results of these studies together with those which have shown histological signs of inflammations [61, 62] and an increase in the gut permeability [63] in patients with AS support the hypothesis that the main bacterial immune response involves mucosal immunity with signs of overt or more commonly occult or asymptomatic intestinal infections by *Klebsiella* microorganisms.

#### 3.2.3. Evidence of Immunological and Microbiological Link between *Klebsiella* and CD

Evidence exists which supports direct and indirect roles for the environmental factors, mostly bacterial, and more specifically involves *Klebsiella* microbes in the pathogenesis of CD. These can be summarized as follows.

- Identical twins of patients with CD are less prone to develop the disease [11], indicating the role for an environmental factor in the development of this disease.
- Increased incidences of CD have been reported among closely living friends of CD patients [64] as well as among small town populations in Southern Italy [65]. Furthermore, a slow increase in the incidence of CD was observed among migrants who are moving from a low to high risk area [66].
- Pretreatment of mice with antibiotics has been shown to alleviate intestinal inflammation in experimental animal models [67, 68], which gives further support to the involvement of gut bacteria in CD.
- Various independent groups have shown that antibodies to *Klebsiella* and/or to crossreactive collagen antigens were elevated more significantly in patients with CD when compared to the control groups [69].
- Evidence of the microbiological links of *Klebsiella* with CD has been mainly based on the isolation of these microbes from large bowel specimens in more than 25% of patients with CD [70]. Moreover, the disease relapses in patients with CD were found to be associated with *Klebsiella oxytoca* colitis [71].

It appears from these data results that unlike rheumatic fever, Lyme disease, and ReA, infections by *Proteus* and *Klebsiella* microbes in RA and AS/CD, respectively, usually occur in occult or subclinical forms, and that antibodies to the causative microbes and crossreactive self-antigens are detected frequently in active patients with these diseases when compared to control groups.

## 4. Exogenous (Microbial) and Endogenous Factors Are the Most Likely Causes of Autoantibody Productions in RA

It should be emphasized that apart from antibodies against the self-antigens, HLA-DR4/1, and collagen fibres, most if not all other autoantibodies such as rheumatoid factor (RF), anticyclical citrullinated protein/peptide antibodies (anti-CCP), and antineutrophil cytoplasmic antibodies (ANCA), which are commonly detected in patients with RA [72, 73], are most likely to be produced as the result of B cell stimulation by exogenous (microbial) agents and/or the effect of some endogenous enzymatic factors.

The interrelation between RF and RA could be explained on the basis of the following findings: firstly, RF can also be detected in increased levels in patients with various viral, bacterial, and parasitic infections [74]. Secondly, RFs can be induced in the mice by polyclonal B cell stimulation with lipopolysaccharides [75]. Thirdly, RFs could be generated by immunization with collagen II antigen-antibody [76] and RF-like immune complexes [77] in experimental mice. Finally, RFs were found to disappear in patients with subacute bacterial endocarditis when the causative microbes, *Streptococcus*, were eradicated by antibiotic therapy [78].

Anti-CCP antibodies, which have been found in early cases of RA [79], can be linked to *Proteus* infections through the effects of peptidyl arginine deiminase (PAD) enzyme on the arginine-containing structures of *Proteus* hemolysin and urease antigens and the counterpart crossreactive HLA-DR4/1 and collagen XI self-antigens, with the production of citrulline-containing compounds which form the main antigenic components of citrullinated proteins [34, 80]. The association of RFs and anti-CCP antibodies with RA explains the existence of a positive correlation between increased levels of these antibodies and the presence of HLA-DR4/1 shared epitope [81] as well as the disease activity and severity in patients with RA [82]. Furthermore, anti-CCP antibodies have also been reported in patients with various microbial infections [83].

Unlike other autoantibodies, ANCAs were recognized in a lower proportion, usually in less than 25% [84, 85] of patients with RA. Apart from RA, however, these antibodies have also been reported in many other diseases, including microbial infections, especially when associated with systemic vasculitis [73]. Moreover, proteinase-3, which is considered as one of the predominant antigens that specifically binds to ANCA, is found to have similarities with some bacterial antigenic profiles [86].

## 5. Molecular Mimicry Hypothesis and Pathogenetic Mechanism in the Development of Microbe-Triggered Rheumatic Diseases

Although the avidity of the interactions between antigenic determinants and specific antibodies is considerably high, these antigen-binding sites can allow epitopes of similar shapes expressed on completely different microbial or animal cells to bind these antibodies, albeit with a lower binding avidity. These so-called crossreactive epitopes are made up of essentially the same amino acid and carbohydrate molecules, and such crossreactions are in fact common and may account for the undesirable production of antibodies against self-molecules which occurs in some autoimmune diseases.

Molecular mimicry or crossreactivity hypothesis proposes that an exogenous substance, mostly produced or possessed by infectious agents, may trigger an immune response against self-antigens. According to this theory susceptible individuals acquire an infection by a microbial agent that has antigenic similarity to self-antigens. As the result, these pathogen-specific antibodies bind to the host structures possessing crossreactive self-antigens and cause tissue damage and disease.

Molecular mimicry has been linked to the pathogenesis of several important diseases, such as rheumatic heart disease [87], multiple sclerosis [88], and type 1 diabetes mellitus [89]. In rheumatic fever carditis, for example, the basic pathogenetic process involves production of antibodies against *Streptococcus* which express high levels of M protein antigens, a molecule that shares structural similarities with those found in the heart valves and endocardial membrane. If antibodies to these bacterial proteins reach high levels, there may be sufficient binding to the host cells possessing these cross-reactive antigens with activation of the complement system and induction of the pathological damages at these sites.

The mechanism of molecular mimicry, however, can also be used in the explanation for the development of RA, AS, and CD after infections by the causative microbes. In AS, for example, after a preliminary gut mucosal activation by *Klebsiella* microbes and production of the secretory anti-*Klebsiella* IgA antibodies, recurrent bouts of subclinical *Klebsiella* infections in the large bowel of susceptible individuals carrying HLA-B27 will lead to production of increased levels of *Klebsiella* IgG antibodies. When the level of these antibodies reaches a certain limit, they will be able to activate the classical cascade of complement system and destroy tissues via the effect of end products of the complement components, mainly C8 and C9, “membrane attack complex.” At the same time, certain activated complement components such as C3a and C5a help in the propagation of the inflammatory process through recruitment (chemoattraction) and activation of the neutrophils and phagocytes with the release of cytotoxic and destructive enzymes by these cells. Other chemoattractants, such as leukotriene B4, can be released by the autoantibody targeted cells. Inflammatory cells are further activated by binding to autoantibody Fc regions and fixed complement C3 fragments on the tissue cells, thus causing further tissue injury via effects of the products of activated inflammatory cells.

There is a requirement for the presence of high levels of anti-*Klebsiella* IgG antibodies in order that classical complement cascades will be activated and this will occur in patients with AS mainly during the active phases of the disease [90, 91]. The same pathogenetic process can also be applied to RA being caused by recurrent bouts of *Proteus* asymptomatic urinary tract infections.

## 6. Conclusions

The aetiopathogenetic mechanism which plays a major role in the causation and development of one group of autoimmune diseases involves interplay between the genetic and environmental factors. Microbes form an important part in the disease causations in most immune-mediated rheumatic diseases, such as rheumatic fever, ReA, Lyme disease, RA, AS, and CD. Molecular mimicry is considered as the basic mechanism which leads to the development of these diseases, in genetically susceptible individuals, where the causative microbe triggers formation of antimicrobial antibodies which could bind the crossreactive self-antigens and cause tissue damages via the effects of activated complement system and the cytotoxic products from recruited inflammatory cells.

## Figures and Tables

**Table 1:** Characteristics of bacterial immune responses in patients with rheumatoid arthritis and ankylosing spondylitis (see [33, 34, 41, 42]).

	Rheumatoid arthritis (RA)	Ankylosing spondylitis (AS)
Disease-triggering bacteria	*Proteus mirabilis*	*Klebsiella pneumoniae*
Bacterial antigens	Hemolysin; Urease	Nitrogenase; Pullulanase
Self-antigens*	HLA-DR4/1; collagen XI	HLA-B27; collagens I, III and IV
Antibody isotypes	IgG	IgA and IgG
Source of bacterial infections	Upper urinary tract	Large bowel
Bacterial isolations	*P. mirabilis* isolated more significantly from urine of active RA patients	*K. pneumonia* microbes are more abundant in the large bowel of active AS patients
Evidence for cytotoxic activities	*Proteus* antibodies are cytotoxic to cells coated with crossreactive self antigens	*Klebsiella* antibodies are cytotoxic to cells coated with crossreactive self antigens
Countries**	England; Ireland; Scotland; USA; Canada; France; Norway; Bermuda; Japan; Taiwan; India; Netherlands; Spain; Russia and Finland	England; Scotland; USA; Canada; Slovakia; China; Netherlands; Turkey; Japan; Finland; Sweden; Mexico; Germany; Taiwan; India; Russia; Spain
Microbial controls^Δ^	*Klebsiella, Escherichia, Yersinia, Salmonella, Chlamydia, Shigella, Pseudomonas, Campylobacters,* and viruses	*Proteus, Escherichia, Yersinia, Salmonella, Streptococci, Borrelia, Pseudomonas, Candida,* and *Campylobacters *
Disease controls^ΔΔ^	AS, systemic lupus erythematosus, sarcoidosis, acute anterior uveitis, spondyloarthropathy	RA, psoriatic arthritis, osteoarthritis, reactive arthritis, systemic lupus erythematosus

* Self-antigens crossreactive with the corresponding bacterial antigens

**Countries with recruited cohort patients showing elevated levels of antibodies against *Proteus* (in RA), *Klebsiella* (in AS) as well as against the corresponding crossreactive self-antigens

^Δ^Microbial agents used as controls but showing no enhanced humoral immune responses in patients with RA or AS

^ΔΔ^Disease controls showing normal immune responses to *Proteus* (in RA) and *Klebsiella* (in AS).
